# Behavioral state differentially regulates input sensitivity and firing rates of motor cortex pyramidal neurons

**DOI:** 10.1186/1471-2202-14-S1-P114

**Published:** 2013-07-08

**Authors:** Paolo Puggioni, Miha Pelko, Mark van Rossum, Ian Duguid

**Affiliations:** 1 Doctoral Training Centre in Neuroinformatics, University of Edinburgh, Edinburgh, UK; 2 Centre for Integrative Physiology, University of Edinburgh, Edinburgh, UK; 3 Institute for Adaptive Neural Computation, University of Edinburgh, Edinburgh, UK

## 

The primary motor cortex (M1) plays a prominent role in the initiation and control of voluntary movements [1], but the cellular mechanisms regulating motor output are not well understood. Due to its direct link with behavior, M1 is an ideal platform to study how brain state and behavior are related to single neuron dynamics. Here we combined *in vivo *patch-clamp recordings and computational modeling to characterize the effects of behavioral state on the membrane potential dynamics and integrative properties of layer 5B pyramidal cells in the awake motor cortex. Furthermore, we injected a range of somatic EPSC-like current pulses during quiet wakefulness and movement to investigate how behavioral state affects input-output transformations in layer 5B excitatory neurons.

We found that during quiet wakefulness L5B cells (n = 24) display moderate firing rates (5.6+-3.5 Hz) and depolarized membrane potential (-52.9+-4.4 mV). In superficial and deep cortical layers of M1, we show that the membrane potential during quiet wakefulness is characterized by slow fluctuations in the delta-band range (2-4 Hz), resulting in a large V_m _standard deviation (3.5+-1.0 mV) that was similar in magnitude to that observed in L2/3 of the barrel cortex of awake mice [2].

By modeling the neuron as a single compartment leaky integrate and fire unit, we estimated that the sum of the average excitatory and inhibitory synaptic conductances were similar in magnitude to the leak conductance (<Ge> = 2.2 nS, <Gi> 3.0 nS, Gl = 5.5 nS) and consistent with values measured in primary visual cortex of awake mice [3].

We classified L5B cells (the main output neurons in M1) into two functional different sub-populations, which either suppressed (3.4+-3.5 Hz, L5Bsupp, n = 10) or enhanced (12.7 +- 5.6 Hz, L5Benh, n = 14) their firing rates during movement. During movement, we observed a global suppression of slow V_m _fluctuations resulting in a decrease of the Vm standard deviation in L5Bsupp cells (2.4 +-0.3 mV, p = 0.002). By injecting small somatic EPSC waveforms, we demonstrated that the decrease in V_m _variance reduced the input sensitivity of L5Bsupp cells during movement. In contrast, L5Benh cells displayed a net depolarization (to -49.1+-4.2 mV, *n *= 14, *p *= 0.0006) due to a moderate increase in average excitatory conductance (ΔG_e _= +0.7 nS) and enhanced Vm fluctuations in the high frequency band (12-50 Hz, Figure 1B). Based on integrate-and-fire simulations, we estimate that during movement excitatory inputs to L5Benh cells increased by +30%, with enhanced fine time-scale correlations (pairwise correlation coefficient of the inputs is ~1% larger). These changes increased the input sensitivity of L5Benh neurons. Thus we find that behavioural state differentially regulates the membrane potential dynamics and integrative properties of discrete subpopulations of output neurons in M1.

Our findings suggest that sensorimotor information is preferentially routed through distinct subpopulations of excitatory neurons during motor behavior. During movement motor output from M1 is controlled by the interplay between a global suppression of slow Vm fluctuations and selective increase in the rate and synchrony of excitatory synaptic inputs.
